# Early Experience with Force-Modulating Tissue Bridges (FMTB, Brijjit) for Skin Closure in Foot and Ankle Surgery: A Single-Surgeon Case Series

**DOI:** 10.1177/24730114261471165

**Published:** 2026-08-26

**Authors:** Nicholas Stamatos, Eric Giza, Alexandra Ernst, Christopher D. Kreulen

**Affiliations:** 1 Department of Orthopaedic Surgery, Department of Orthopaedic Surgery, University of California, Davis, Sacramento, CA, USA

**Keywords:** force-modulating tissue bridge, FMTB, Brijjit, wound complications, foot and ankle surgery, skin closure, tension offloading

## Abstract

**Background:**

Wound complications following foot and ankle surgery remain challenging, with rates of 4% to 36% depending on procedure complexity. Mechanical tension contributes to dehiscence through mechanotransduction pathways. The force-modulating tissue bridge (FMTB) reduces tissue-interface pressure from approximately 4000 mm Hg (sutures) to 20 mm Hg. Randomized controlled trial evidence in breast surgery demonstrates 89% relative risk reduction in wound complications; however, data specific to foot and ankle surgery are lacking.

**Methods:**

Retrospective case series of consecutive foot and ankle operations closed with FMTBs by a single fellowship-trained surgeon (March-October 2025). Primary outcome was any wound complication within 90 days. Each event was retrospectively classified using a framework consistent with ISO 14155:2020 to distinguish FMTB application-site reactions from other causes.

**Results:**

Twenty-nine patients were included (mean age 57.2 years; 66% male; mean body mass index [BMI] 30.9). Most procedures involved the ankle (62%), with complex reconstructions predominating (mean tourniquet time 104 minutes). The 90-day wound complication rate was 41.4% (12/29; 95% CI: 25.5%-59.3%), consistent with published rates for complex foot and ankle procedures when all wound events are captured. Major complications occurred in 3 patients (10.3%); all were attributable to severe patient-level confounding factors (pre-existing osteomyelitis, morbid obesity with heavy smoking history, and chlorhexidine allergy) rather than the FMTB device. After applying this classification framework, FMTB application-site skin reactions occurred in 2 patients (6.9%). Notable among those without complications was 1 patient with BMI 48.5 and only 1 remaining lower limb.

**Conclusion:**

This case series describes the feasibility and acceptable tolerance of FMTB closure in a consecutive cohort of foot and ankle procedures spanning the full clinical spectrum. The 90-day wound complication rate (41.4%, 95% CI 25.5%-59.3%) is consistent with published rates for high-complexity foot and ankle surgery when all wound events are captured; device-application-site skin reactions occurred in 6.9% of patients (2/29) and resolved with local wound care. These descriptive data do not establish comparative efficacy. Prospective controlled studies are needed to determine whether FMTB-mediated tension offloading provides incremental wound-healing benefit over conventional closure in this anatomically challenging region.

**Level of Evidence:**

Level IV, therapeutic, retrospective case series.

## Introduction

Wound complications represent a significant challenge in foot and ankle surgery. The anatomic characteristics of the lower extremity—including thin soft tissue coverage, tenuous end-arterial blood supply, dependent positioning, and exposure to weight-bearing stress—create an environment prone to wound healing difficulties. Published surgical site infection (SSI) rates in foot and ankle surgery average 4.4% across procedures,^1^ but wound complication rates vary substantially based on procedure complexity: 7% to 30% for ankle fracture fixation,^2,3^ 19.7% to 28% for total ankle arthroplasty (TAR),^4-6^ and up to 36% for Charcot reconstruction, with one systematic review of 1116 feet reporting an aggregate complication rate of 36% that encompasses dehiscence, deep infection, hardware failure, and reulceration.^7,8^

Patient risk factors substantially affect wound outcomes. Diabetes mellitus is associated with an 8-fold increase in superficial wound infection following tibiotalocalcaneal arthrodesis,^9^ obesity (body mass index [BMI] 30 or greater) is associated with a 3-fold higher wound complication rate,^10^ and tourniquet time of 90 minutes or more has been independently associated with wound complications in foot and ankle surgery (adjusted odds ratio 7.02, 95% CI 2.77-19.32),^11^ with elevated risk also observed in anterior ankle incisions specifically.^12^ Furthermore, wound dehiscence has been associated with a 21-fold increase in infection risk, emphasizing the clinical significance of maintaining wound integrity.^13^

Mechanical tension across incisions has been identified as a key factor contributing to wound complications. Through mechanotransduction pathways—including TGF-β1/Smad, FAK-ERK, and YAP/TAZ signaling—sustained mechanical stress induces fibrotic responses, impairs cell migration, disrupts collagen organization, and compromises microcirculation.^14,15^ Traditional transcutaneous sutures generate compressive stresses of approximately 4000 mm Hg at the tissue-suture interface, which can exceed capillary perfusion pressure and contribute to tissue ischemia.^16^

Tension-offloading closure devices represent an emerging strategy to address this biomechanical challenge. The force-modulating tissue bridge (FMTB [Brijjit; BRIJ Medical, Inc]) is a tension-offloading skin closure device that distributes closure forces across a wound area while maintaining wound approximation. Finite element analysis demonstrates that FMTBs reduce tissue-interface pressure to approximately 20 mm Hg—a 200-fold reduction compared to sutures—while providing 30% sustained strain reduction over a 10-day treatment period.^16,17^ This level of strain reduction with induction of tissue eversion can be indicative of up to 100% tension reduction at the wound interface.

Existing clinical evidence for the FMTB derives from breast surgery. A randomized controlled trial in bilateral breast reduction demonstrated that T-junction dehiscence occurred in only 1/34 FMTB-treated incisions (2.9%) versus 11/34 standard closure incisions (32.4%), representing an 89% relative risk reduction (*P* < .01), with a number needed to treat of 3 breasts (approximately 2 patients).^18^ A matched cohort study confirmed these findings, with major wound events reduced by 89% and a number needed to treat of only 8.^19^ Safety data from >35 000 cases demonstrate no reported allergic reactions to the device.^20^

Whether the FMTB benefit observed in breast surgery translates to the thicker, more poorly vascularized, dependently positioned, and weight-bearing foot and ankle soft tissue envelope is unknown. The distinct biomechanical and vascular profile of the lower extremity, combined with the higher comorbidity burden of foot and ankle surgical patients, warrants specific investigation rather than extrapolation. We therefore sought to describe our early experience with FMTB closure in a consecutive series of foot and ankle procedures.

## Methods

### Study Design and Population

We evaluated a retrospective case series of all consecutive foot and ankle operations in which FMTBs were used for skin closure. All procedures were performed by a single fellowship-trained foot and ankle surgeon at an academic medical center between March 5, 2025, and October 29, 2025, corresponding to the surgeon’s initial adoption and subsequent routine use of the device. FMTB devices were applied for primary skin closure in all consecutive open foot and ankle procedures performed by the senior author (CK and EG) during the study period, without selection on the basis of patient risk profile, procedure type, or anticipated complication risk. The only exclusion criterion was percutaneous procedures without an open incision. All 29 analytic patients had complete 90-day follow-up. One additional patient underwent FMTB-assisted closure during the study period but died of unrelated causes prior to completion of 90-day wound surveillance and was excluded from the analysis (final analytic n = 29). This study was approved as exempt by the institutional review board under the institution’s protocol for retrospective analysis of de-identified surgical outcomes data (45 CFR [Code of Federal Regulations] 46.104). Informed consent was waived given the retrospective design and exclusive use of de-identified records. All clinical photographs represent de-identified close-up images of surgical sites containing no patient-identifiable features. This study follows the PROCESS guidelines for reporting surgical case series.^21^

The cohort intentionally included the full spectrum of clinical scenarios to assess device utility: primary elective procedures, complex revisions, urgent trauma reconstruction, and rescue closure for failed prior surgery.

### Data Collection

Data were abstracted from operative reports, anesthesia records, and postoperative clinic notes using a standardized case report form. Variables collected included demographics (age, sex, BMI), comorbidities (diabetes mellitus, smoking status, peripheral neuropathy, peripheral vascular disease, immunosuppression, connective tissue disorders), operative details (anatomic zone, procedure type, tourniquet time, FMTB device count), and postoperative course.

### Outcome Definitions

The primary outcome was any wound complication within 90 days of surgery, defined as a composite of wound dehiscence requiring change in wound care; surgical site infection per US Centers for Disease Control and Prevention (CDC) definitions^22^ requiring antibiotics or operative intervention; hematoma or seroma requiring drainage; wound necrosis; unplanned visit for wound concerns; or return to operating room for wound complications.

### Complication Adjudication

Each potential wound complication was retrospectively classified using a framework consistent with ISO 14155:2020 (Clinical investigation of medical devices for human subjects—Good clinical practice) and MEDDEV 2.7/3 Rev 3, with events categorized as Not Related, Possibly Related, Probably Related, or Causally Related to the FMTB device. The framework was applied to all primary outcome events (dehiscence, surgical site infection, and skin findings at the device application site). Each event was reviewed against available clinical documentation from operative reports and postoperative clinic notes, with classifications assigned by applying the criteria below. A complication was classified as device-related (Probably or Causally Related) when (1) the finding was localized to the FMTB application zone rather than the primary surgical incision proper, (2) there was a documented temporal relationship to FMTB exposure, and (3) no alternative cause (eg, patient comorbidity, splint or dressing pressure, surgical technique factor, prep solution reaction) provided a more parsimonious explanation. Surgical site infections were classified per the CDC National Healthcare Safety Network (NHSN) criteria as superficial incisional, deep incisional, or organ/space. Wound dehiscence was attributed to the primary surgical incision unless skin separation occurred specifically at the FMTB-skin interface in the absence of incisional dehiscence.

By way of example, a small closed blister observed at the lateral fifth metatarsophalangeal joint of 1 patient 21 days postoperatively—anatomically remote from both the surgical incisions and the FMTB application zone, and temporally and causally attributable to a splint that was documented to not be maintaining neutral foot position—was classified as Not Related to the FMTB device and was not included in the wound-complication count.

### Surgical Technique

**Figure 1.**
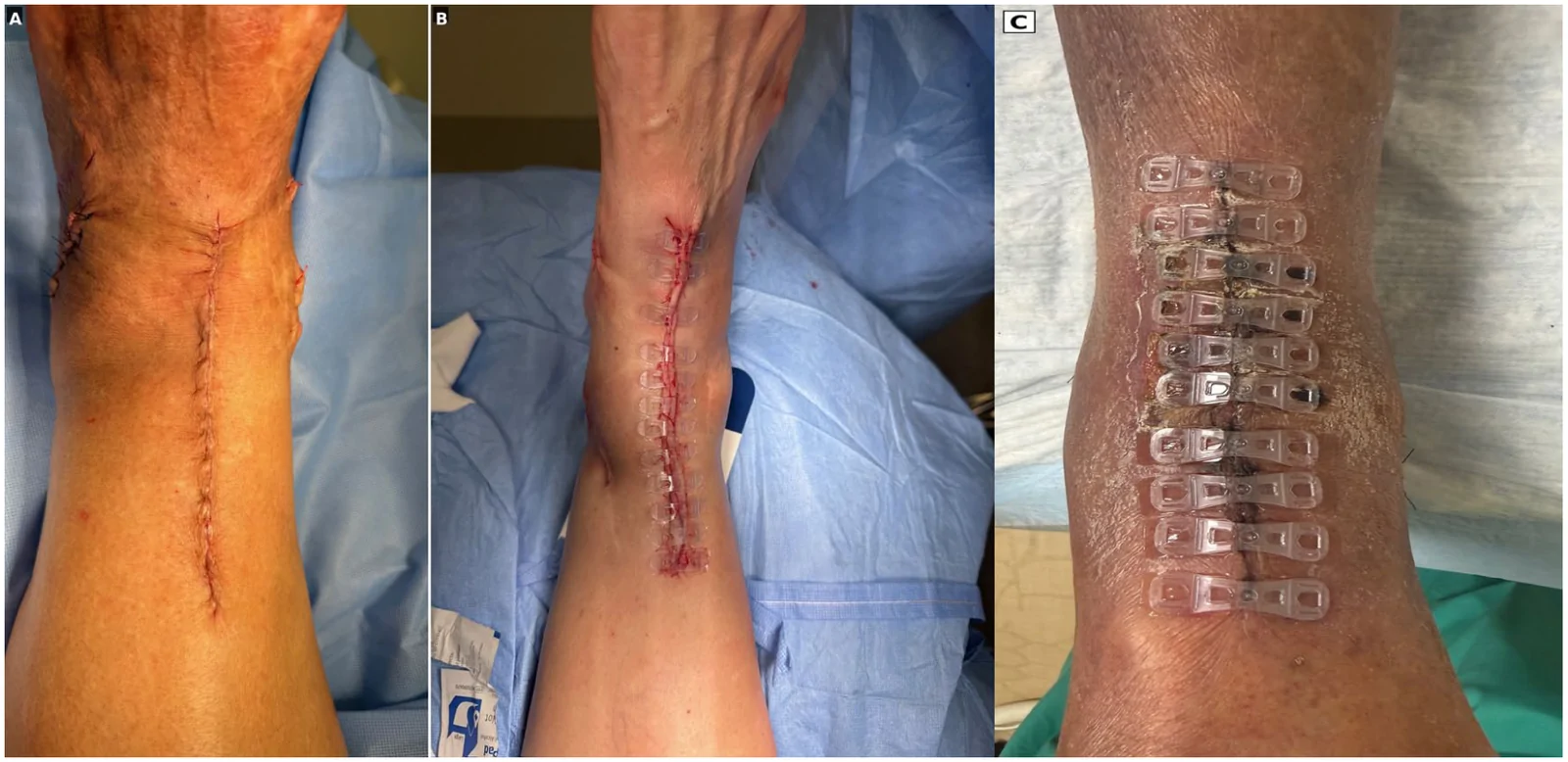
Intraoperative FMTB (Brijjit) application technique. (A) Anterior ankle incision closed with layered absorbable sutures prior to FMTB application. (B) FMTB devices (Brijjit BP100) applied perpendicular to the incision, spaced approximately every 2 cm along the wound length. (C) Completed application demonstrating full-length wound coverage with multiple devices. FMTB, force-modulating tissue bridge.

Layered closure was performed with deep absorbable sutures (Vicryl and barbed Monocryl) for fascial and subcutaneous layers. Skin closure was achieved using FMTB devices (Brijjit BP100), with 1 to 4 packs used per case (6 devices per pack) applied perpendicular to the incision, spaced approximately every 2 cm along the wound length (Figure 1; Supplementary Video 1). Devices were removed at a postoperative visit (14-21 days). At device removal, the FMTB adhesive pad commonly lifted a thin layer of stratum corneum with intact underlying epithelium and the primary incision unaffected; this isolated stratum corneum lift, occurring uniformly across the adhesive footprint and without surrounding inflammation, was anatomically and clinically distinct from focal blistering at the device application site adjacent to the incision, and was not classified as a wound complication or device-related skin reaction.

### Statistical Analysis

Continuous variables are summarized as mean ± SD with range, and categorical variables as count and percentage. Wound complication rates and final outcome distributions are reported with Wilson score 95% CIs, which provide improved coverage over normal-approximation intervals for small samples and proportions near 0 or 1. Given the small sample size and descriptive intent of this case series, no inferential hypothesis testing was performed; subgroup observations are presented descriptively only. Analyses were performed in Python (version 3.11) using the statsmodels and scipy.stats libraries.

## Results

### Patient Characteristics

Twenty-nine patients underwent FMTB-assisted closure during the study period. The cohort encompassed primary elective procedures (n = 23, 79%), complex revisions (n = 4, 14%), urgent trauma reconstruction (n = 1, 3%), and rescue closure for failed prior surgery (n = 1, 3%). Complete patient-level data are provided in Supplementary Table 1.

Mean age was 57.2 ± 18.9 years (range 14-77), with 19 males (66%) and 10 females (34%). Mean BMI was 30.9 ± 8.5 (range 20.1-51.0), with 11 patients (38%) classified as obese (BMI 30 or greater). Diabetes or prediabetes was present in 3 patients (10%), peripheral neuropathy in 4 (14%), and chronic immunosuppression in 2 (7%). One patient had Ehlers-Danlos syndrome. ASA classification was predominantly 2 (48%) or 3 (41%).

### Operative Details

**Figure 2.**
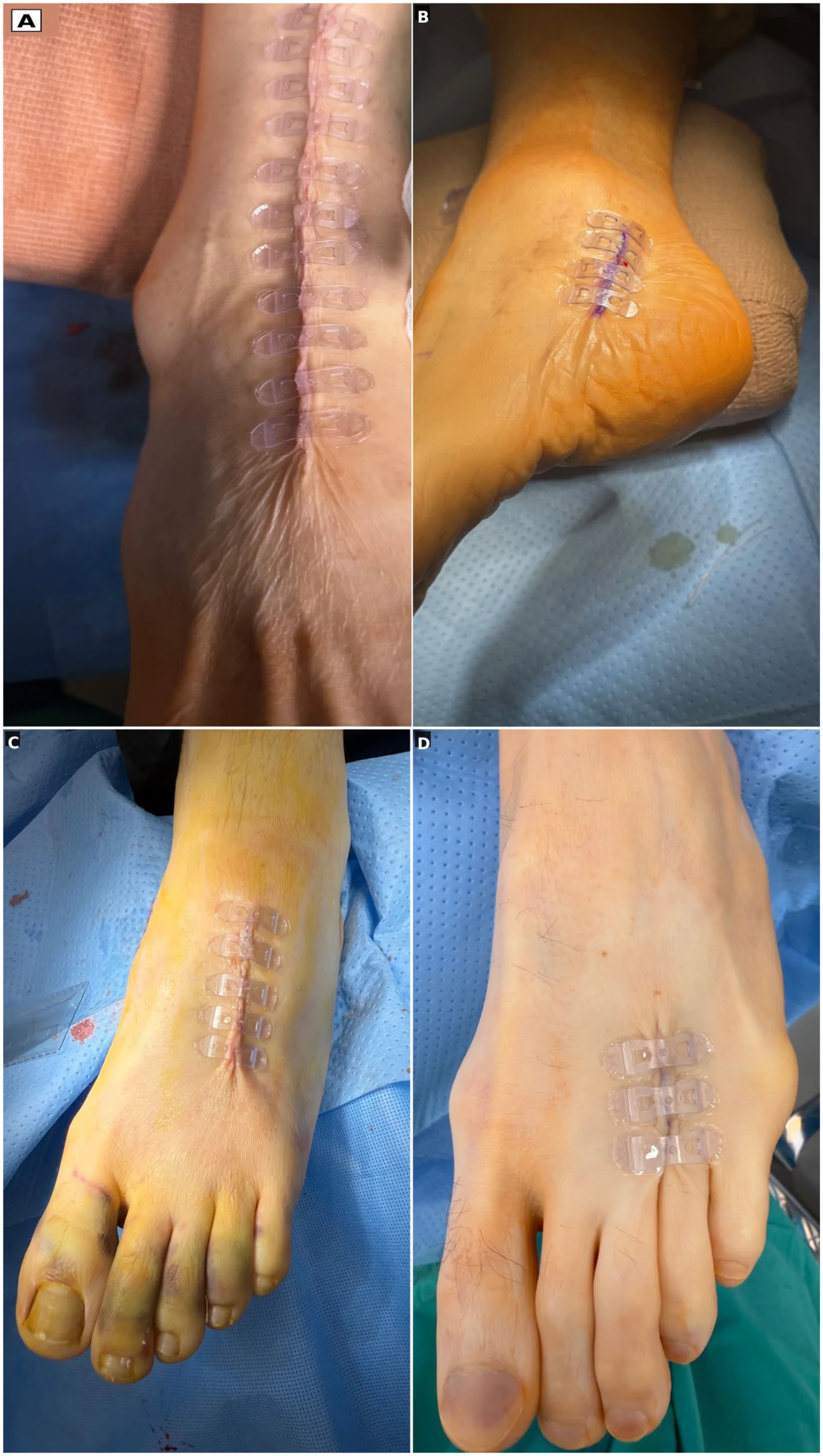
FMTB application across anatomic zones. (A) Anterior ankle incision for total ankle replacement. (B) Hindfoot incision for subtalar or tibiotalocalcaneal fusion. (C) Midfoot dorsal incision for Lisfranc or tarsometatarsal reconstruction. (D) Forefoot incision for first metatarsophalangeal joint procedure. FMTB, force-modulating tissue bridge.

The most common anatomic zone was the ankle (18 patients, 62%), followed by midfoot (6, 21%), hindfoot (3, 10%), and forefoot (2, 7%) (Figure 2). The procedure mix included total ankle arthroplasty (n = 8, 28%), tibiotalocalcaneal arthrodesis (n = 3, 10%), other ankle procedures including arthrodesis, open reduction and internal fixation, and revision (n = 7, 24%), midfoot procedures including Lisfranc/tarsometatarsal reconstruction and arthrodesis (n = 6, 21%), hindfoot procedures including revision (n = 3, 10%), and forefoot procedures (n = 2, 7%). Mean tourniquet time was 104 ± 41 minutes (range 27-182), with 16/24 (67%) having tourniquet time of 90 minutes or more (among cases with documented tourniquet use). FMTB device count ranged from 1 to 4 per case (mean 1.5). Patient demographics, comorbidities, and operative details are summarized in Table 1.

**Table 1.** Patient Demographics and Baseline Characteristics (n = 29).^a^

Characteristic	Value
Age, y, mean ± SD (range)	57.2 ± 18.9 (14-77)
Male sex, n (%)	19 (66)
BMI, mean ± SD (range)	30.9 ± 8.5 (20.1-51.0)
Obese (BMI ≥30), n (%)	11 (38)
Comorbidities, n (%)	
Diabetes/prediabetes	3 (10)
Peripheral neuropathy	4 (14)
Immunosuppression	2 (7)
ASA classification, n (%)	
ASA 1	3 (10)
ASA 2	14 (48)
ASA 3	12 (41)
Anatomic zone, n (%)	
Ankle	18 (62)
Midfoot	6 (21)
Hindfoot	3 (10)
Forefoot	2 (7)
Operative Details	
Tourniquet time, min, mean ± SD (range)	104 ± 41 (27-182)
Tourniquet time ≥90 min, n (%)	16/24 (67)
FMTB devices per case, mean ± SD	1.5 ± 0.7

Abbreviations: ASA, American Society of Anesthesiologists; BMI, body mass index; FMTB, force-modulating tissue bridge.

a Tourniquet values calculated among cases with documented tourniquet time greater than 0 (n = 24). Tourniquet time was not recorded in 4 cases; 1 case had no tourniquet.

### Primary Outcome

All 29 patients had complete 90-day follow-up. The 90-day wound complication rate was 41.4% (12/29; 95% CI: 25.5%-59.3%). The 30-day complication rate was 34.5% (10/29; 95% CI: 19.9%-52.7%). Under the causality framework described in Methods, all dehiscences and deep surgical site infections were classified as Not Related to the FMTB device, with attribution to procedure-related and patient-level factors documented in the clinical record; device-application-site events were classified separately and are reported in the Device Tolerance section. Wound complication rates and final outcome classifications are summarized in Table 2.

**Table 2.** Wound Complications and Outcomes.^a,b^

Outcome	n/N (%)	95% CI
Primary outcome		
90-d wound complication	12/29 (41.4)	25.5%-59.3%
Secondary outcomes		
30-d wound complication	10/29 (34.5)	19.9%-52.7%
Dehiscence	8/29 (27.6)	14.7%-45.7%
Deep SSI	3/29 (10.3)	3.6%-26.4%
Return to OR for wound	2/29 (6.9)	1.9%-22.0%
Device tolerance		
FMTB application-site skin reaction	2/29 (6.9)	1.9%-22.0%
Final outcome classification		
No complication	17 (58.6)	40.7%-74.5%
Minor complication	8 (27.6)	14.7%-45.7%
Major complication	3 (10.3)	3.6%-26.4%
Healing (ongoing)	1 (3.4)	0.6%-17.2%

Abbreviations: FMTB, force-modulating tissue bridge; OR, operating room; SSI, surgical site infection.

a CIs are based on the Wilson score method. 95% Wilson score CIs are reported for all primary, secondary, device-tolerance, and final outcome classification rows.

b After application of the classification framework (see Methods); classified as Probably or Causally Related to the FMTB device.

### Specific Complication Types

Dehiscence occurred in 8 patients (27.6%; 95% CI: 14.7%-45.7%). Five were minor superficial separations (1-2 mm openings) managed with local wound care alone; the remaining 3 were associated with deep surgical site infection (described below) and required operative intervention as part of their infection management. Deep surgical site infection occurred in 3 patients (10.3%; 95% CI: 3.6%-26.4%), all requiring operative intervention. Return to operating room specifically for wound complications occurred in 2 patients (6.9%; 95% CI: 1.9%-22.0%).

### Device Tolerance

Six patients (20.7%) had skin findings initially flagged for review. Applying the causality framework described in Methods produced the following classifications: 1 patient developed a blister classified as Not Related to FMTB (chlorhexidine surgical prep allergy, with anatomic distribution consistent with prep solution contact); 2 patients with forefoot procedures at the medial first metatarsal developed maceration patterns classified as Possibly Related (one had Ehlers-Danlos syndrome, suggesting an underlying connective tissue contribution); 1 had minor superficial drainage treated with a short oral antibiotic course, classified as Not Related to the FMTB. After applying this framework, FMTB application-site skin reactions classified as Probably Related occurred in only 2 patients (6.9%; 95% CI: 1.9%-22.0%): a 14-year-old with morbid obesity (BMI 51) undergoing hindfoot revision surgery who developed swelling-related blistering, and a 67-year-old undergoing a complex ankle reconstruction with 150-minute tourniquet who developed blistering temporally associated with dressing changes; both healed with local wound care. No allergic reactions to the FMTB occurred in our series.

### Final Outcomes

**Figure 3.**
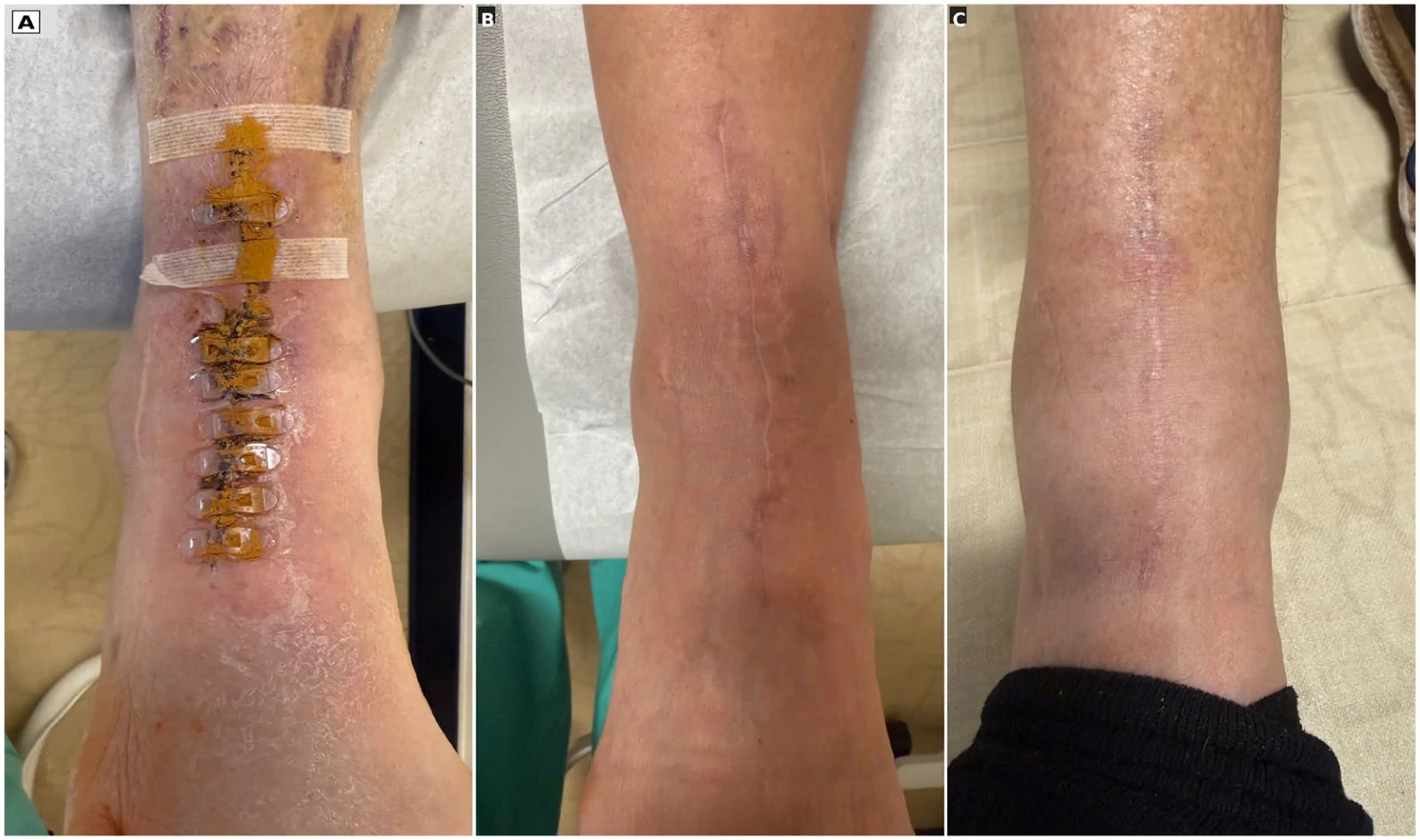
Wound healing progression following FMTB-assisted closure of an anterior ankle incision. (A) Early postoperative appearance with FMTB devices in situ and betadine dressing at the first follow-up visit. (B) Healed incision at approximately 6 weeks following device removal. (C) Final scar appearance at 3 months demonstrating complete wound maturation. FMTB, force-modulating tissue bridge.

At final follow-up, 17 patients (58.6%; 95% CI: 40.7%-74.5%) had no wound complications, 8 (27.6%; 95% CI: 14.7%-45.7%) had minor complications that healed conservatively (including the 2 device-related skin reactions), 3 (10.3%; 95% CI: 3.6%-26.4%) had major complications unrelated to device tolerance, and 1 (3.4%; 95% CI: 0.6%-17.2%) had healthy granulation tissue at 3 months following rescue closure (Figure 3). The 3 major complications occurred in a patient with morbid obesity (BMI 40) and a 37-pack-year smoking history who developed periprosthetic joint infection, a patient whose chlorhexidine surgical prep allergy created a blister serving as an infection nidus leading to deep SSI, and a patient with pre-existing chronic osteomyelitis, 4 prior surgeries, and severe noncompliance who developed SSI requiring prolonged wound vacuum-assisted closure therapy. None of these complications were related to the FMTB device.

### Instructive Cases

**Figure 4.**
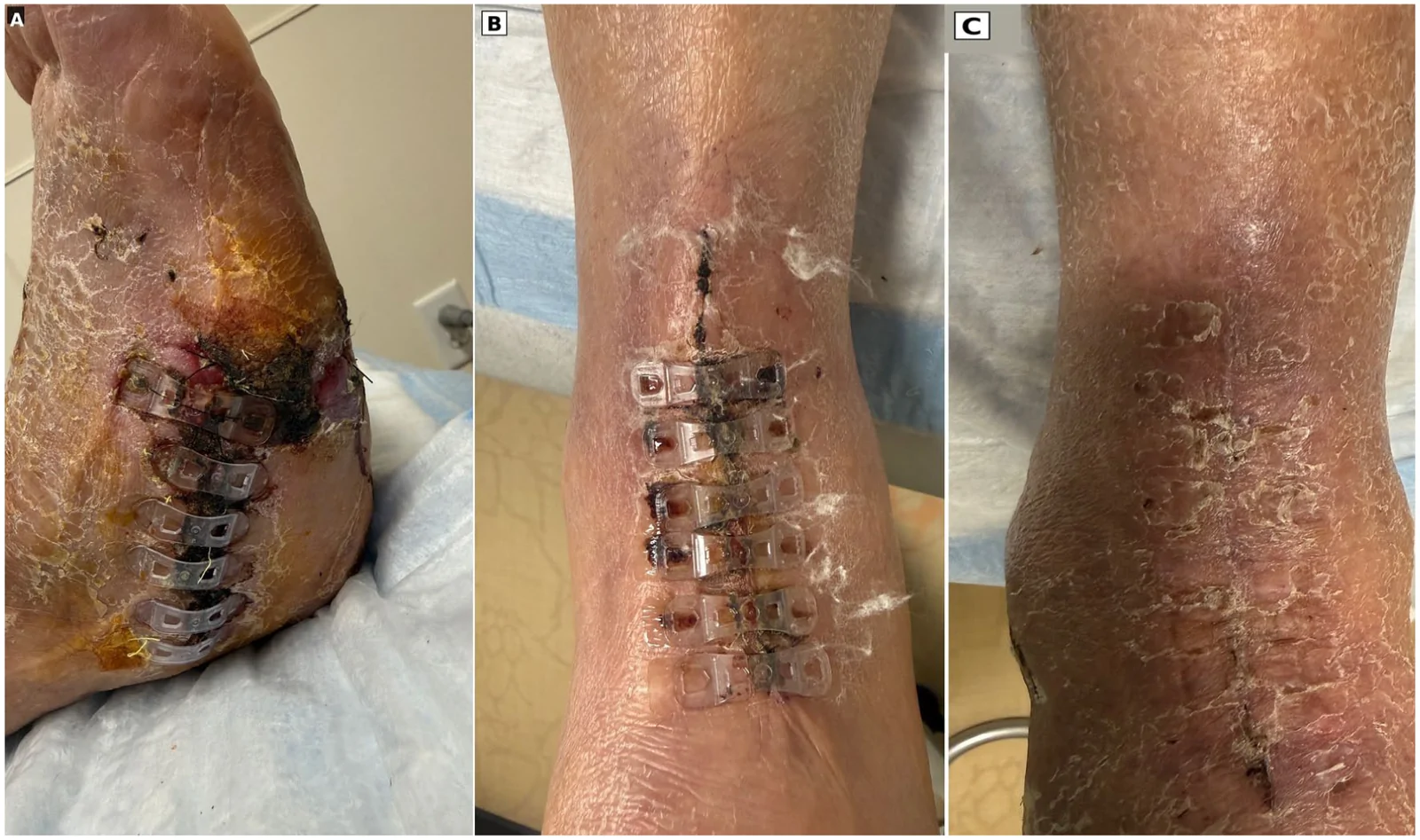
FMTB application in high-risk patients with challenging wound environments. (A) Hindfoot closure in a patient with severely compromised soft tissue envelope and prior surgical scarring. (B) Anterior ankle closure in a patient with marked peri-incisional erythema and tissue edema. (C) Healed wound in a high-risk patient demonstrating satisfactory outcome despite hostile soft tissue conditions. FMTB, force-modulating tissue bridge.

**Figure 5.**
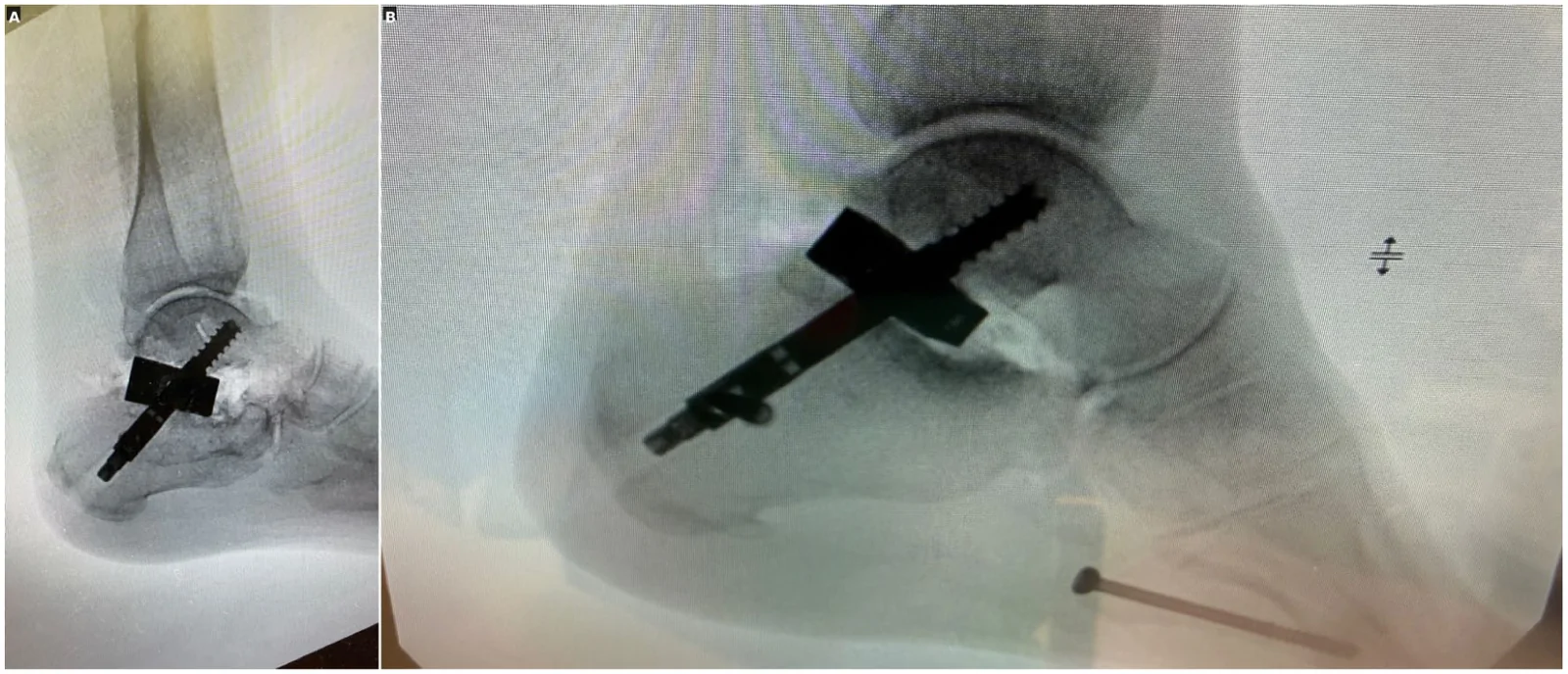
Intraoperative fluoroscopic images demonstrating subtalar arthrodesis hardware. (A) Lateral view of the subtalar arthrodesis construct with screw fixation. (B) Anteroposterior view of the same construct, illustrating the internal fixation underlying the hindfoot incision closed with FMTB devices.

Several patients with extreme risk profiles achieved excellent wound healing (Figure 4). A 23-year-old with morbid obesity (BMI 48.5), only 1 remaining lower limb (contralateral above-knee amputation), and recent deep vein thrombosis healed without complication following subtalar fusion (Figure 5). A 75-year-old with heart failure (ejection fraction 30%), chronic kidney disease, peripheral neuropathy, and chronic prednisone use also healed without wound issues. A patient with documented adhesive allergy (history of hive reactions to dressings after prior surgery) underwent a complex total ankle replacement reconstruction and tolerated 21 days of FMTB application without skin reaction or wound complication. A 27-year-old man with spastic diplegic cerebral palsy and dense motor deficits underwent a complex tibiotalocalcaneal fusion with deformity correction (134-minute tourniquet) and healed without wound complication through 6 months of follow-up, despite the dynamic incisional tension generated by underlying spasticity. Conversely, 1 patient with pre-existing chronic osteomyelitis, 4 prior surgeries, and severe noncompliance developed wound complications despite FMTB closure, illustrating that tension-offloading alone cannot overcome all severe biologic and behavioral barriers to wound healing.

## Discussion

To our knowledge, this case series represents the first published report of FMTB use in foot and ankle surgery. Although the FMTB has been clinically evaluated in breast procedures,^18,19^ the lower extremity presents distinct biomechanical and vascular challenges that warranted specific investigation.

The overall 90-day wound complication rate of 41.4% must be interpreted in the context of our case mix and the distinction between device-related and non–device-related events. This cohort intentionally included complex cases: mean tourniquet time exceeded 100 minutes (above the 90-minute threshold associated with increased wound complication risk^11^), 38% of patients were obese (associated with 3-fold higher wound complication rate^10^), and the cohort included rescue closures and patients with pre-existing chronic osteomyelitis. All 3 major complications were attributable to severe patient-level confounding factors—morbid obesity with extensive smoking history, chlorhexidine allergy creating an infection nidus, and pre-existing osteomyelitis with non-compliance—rather than the FMTB device itself. Published wound complication rates for similar complex procedures range from 20% to 41%: TAR via an anterior approach demonstrates rates between 19.7% and 28%,^5,6^ and Charcot reconstruction averages 36%, with internal fixation reaching 41%.^7,8^

Importantly, our reported dehiscence rate of 27.6% reflects comprehensive capture of all wound separations, including minor superficial dehiscences managed conservatively with local wound care. The foot and ankle literature variably defines and reports wound complications, creating substantial heterogeneity in published rates. Studies counting only major complications requiring operative intervention report rates of 3% to 8%, whereas those comprehensively documenting all wound events in anterior ankle surgery report rates of 19% to 33%.^23^ For example, Raikin et al^23^ documented 25% minor wound complications (healed with local care or oral antibiotics) plus 8% major complications (requiring operative intervention) in 106 total ankle arthroplasties—a 33% total rate comparable to our findings. The anterior ankle incision carries inherently higher risk because of poor soft tissue coverage, limited blood supply, and tension during dorsiflexion, which likely accounts for the higher rates observed in both our series and comparable literature.

This finding is further consistent with broader foot and ankle wound-event literature when all events—including minor superficial separations managed conservatively—are captured. Wiewiorski et al^11^ reported a 16.9% wound complication rate in 295 elective foot and ankle procedures in a generally lower-risk cohort, whereas Macera et al^24^ documented a 36.0% overall complication rate in 378 consecutive ankle open reduction and internal fixation procedures (4.5% minor + 31.5% major). In ankle arthrodesis, Reb et al^5^ reported delayed wound healing in 19.7% of 304 patients via anterior incision. Lehtonen et al^25^ specifically examined closure technique in ankle fracture ORIF and reported local wound-related complications in 10.6% of 94 patients regardless of whether suture or staple closure was used. The substantial variability in published rates—ranging from 3% to >40% for the same procedure type—reflects definitional heterogeneity rather than true outcome variation, with studies counting only operative-revision events reporting the lowest rates and studies comprehensively capturing all wound events reporting rates more consistent with our findings.

A key finding is acceptable device tolerance. After applying this classification framework, FMTB application-site skin reactions classified as Probably Related occurred in 6.9% of patients (2/29). For comparative context, contact dermatitis rates for liquid skin adhesives in orthopaedic surgery have been reported between 2.8% and 7%,^26^ and Lehtonen et al^25^ reported local wound-related complications in 10.6% of patients regardless of whether suture or staple closure was used for ankle fracture ORIF. No allergic reactions to the FMTB occurred in our series. Our observation that a patient with documented adhesive allergy (atopic background with hive reactions to dressings after prior surgery) tolerated 21 days of FMTB application without skin reaction is consistent with Wall et al’s^19^ finding that patients with “adhesive allergies” did not react to FMTB devices. This may reflect the fact that cyanoacrylates do not cross-react with methacrylates, which are responsible for most adhesive-related contact allergies.^27^ Direct comparison of FMTB skin-reaction rates with suture- or staple-only closure is limited by definitional heterogeneity across the published literature and the absence of a matched control arm in our series.

The outcomes in several extremely high-risk patients are consistent with mechanotransduction principles. By reducing tissue-interface pressure from 4000 to 20 mm Hg, FMTBs theoretically modulate pro-fibrotic pathways including TGF-β1/Smad and FAK-ERK signaling toward optimal healing. Beyond these molecular pathways, the reduction in compressive forces may be clinically significant at a more fundamental level: suture-generated pressures of 4000 mm Hg far exceed capillary perfusion pressure, inducing focal tissue ischemia and necrosis at the suture-tissue interface—analogous to the “railroad track” marks seen with transcutaneous sutures externally. Internally, significant foci of necrosis may progress to wound dehiscence (typically during week 2), whereas smaller foci may heal but serve as a nidus for infection.^14-16^ We cannot attribute these outcomes solely to the FMTB without a control group; however, the observation that a patient with BMI greater than 48 and a patient on chronic immunosuppression tolerated the device through routine wound healing is consistent with a hypothesis that tension offloading may provide benefit in challenging soft tissue environments—a hypothesis that requires prospective comparative testing.

Other tension-offloading approaches have demonstrated benefit in foot and ankle surgery. Negative pressure wound therapy on closed incisions reduces deep SSI by 40% and dehiscence by 59% in orthopaedic trauma.^28^ Adhesive suture retention devices in diabetic amputations show 81% dehiscence reduction with substantial cost savings.^29^ The FMTB provides active tension offloading without equipment burden, maintains adherence for weeks, and requires no percutaneous puncture.

Based on this early experience, FMTB application appears technically feasible across the full spectrum of foot and ankle procedures, including high-tension anterior ankle incisions, revision surgery with compromised soft tissue, and patients with multiple wound-healing risk factors. Whether FMTB-mediated tension offloading provides incremental benefit over conventional closure in any of these settings remains undefined and requires prospective comparative evaluation. The forefoot findings suggest that high-friction, weight-bearing areas like the medial first metatarsal may warrant protocol modifications such as earlier device removal or additional protective measures.

### Limitations

This study has limitations inherent to retrospective case series. Without a matched control group closed by conventional technique, we cannot establish comparative effectiveness; this is the fundamental constraint on inference from these data. The consecutive nature of the cohort introduces substantial procedural heterogeneity—spanning total ankle arthroplasty, tibiotalocalcaneal arthrodesis, ankle fracture fixation, Lisfranc reconstruction, hindfoot revision, and forefoot procedures—which limits inference beyond the aggregate-level observations reported. The small sample size limits subgroup analyses. Single-surgeon experience may limit generalizability. A comparative analysis of wound outcomes against historical controls closed with conventional technique is in planning and will be the subject of a future report. The FMTB is applied as a skin-closure adjunct following standard layered subcutaneous and deep dermal closure rather than as a substitute for meticulous deep closure, and the absence of a same-surgeon suture-only control closed with equivalent deep-closure technique precludes any direct comparison with conventional closure.

## Conclusion

This case series provides the first published data on FMTB use in foot and ankle surgery. The device was feasible to apply across the full spectrum of foot and ankle procedures and was acceptably tolerated, with FMTB application-site skin reactions in 6.9% of patients and no allergic reactions despite documented adhesive sensitivity in 1 patient. The overall 90-day wound complication rate (41.4%, 95% CI 25.5%-59.3%) is consistent with published rates for high-complexity foot and ankle surgery when all wound events are captured. These descriptive data do not support a routine clinical recommendation. Prospective comparative studies against conventional closure are needed to determine whether FMTB-mediated tension offloading provides incremental wound-healing benefit in foot and ankle surgery.

## Supplemental Material

sj-pdf-1-fao-10.1177_24730114261471165 – Supplemental material for Early Experience with Force-Modulating Tissue Bridges (FMTB, Brijjit) for Skin Closure in Foot and Ankle Surgery: A Single-Surgeon Case Series Supplemental material, sj-pdf-1-fao-10.1177_24730114261471165 for Early Experience with Force-Modulating Tissue Bridges (FMTB, Brijjit) for Skin Closure in Foot and Ankle Surgery: A Single-Surgeon Case Series by Nicholas Stamatos, Eric Giza, Alexandra Ernst and Christopher D. Kreulen in Foot & Ankle Orthopaedics

## Appendix

**Supplementary Table 1.** Individual Patient Characteristics and Outcomes (n = 29).^a^

Patient No.	Age	Sex	BMI	Key Comorbidities	Zone	Procedure Type	TQ (min)	FMTB #	Wound Comp.	Comp. Type	Final Outcome
1	68	M	—	—	Ankle	TAR	—	—	N	—	No complication
2	75	M	37.3	PreDM; Neuropathy; Chronic prednisone; Obese (BMI 37.3)	Midfoot	Midfoot procedure	75	1	N	—	No complication
3	48	F	20.1	Neuropathy; Smoking (Former)	Ankle	Revision	160	—	N	—	No complication
4	63	M	37.5	Obese (BMI 37.5)	Ankle	TAR	—	—	Y	Dehiscence	Minor
5	69	F	35.0	Obese (BMI 35.0)	Ankle	TAR	120	1	N	—	No complication
6	65	F	24.6	—	Ankle	Ankle procedure	114	1	N	—	No complication
7	45	F	23.7	—	Ankle	TAR	112	4	N	—	No complication
8	59	F	29.2	SLE on tacrolimus; cirrhosis; neuropathy; smoking (former); coagulopathy	Midfoot	Midfoot procedure	47	2	Y	Postop bleeding, ED visit POD 0	Minor
9	73	M	—	—	Ankle	Ankle procedure	103	2	N	—	No complication
10	72	M	25.7	—	Ankle	TAR	135	1	N	—	No complication
11	72	M	24.9	Smoking (former)	Ankle	Ankle procedure	95	2	N	—	No complication
12	61	F	26.3	Ehlers-Danlos syndrome	Forefoot	Bunion correction	60	1	Y	Dehiscence	Minor
13	77	M	25.8	—	Ankle	TAR	144	1	Y	Superficial incisional concern, antibiotics	Minor
14	41	M	27.5	Chronic osteomyelitis; multiple prior surgeries	Ankle	Ankle procedure	182	2	Y	Dehiscence; Deep SSI	Major
15	31	F	21.6	—	Forefoot	Bunion correction	49	1	Y	Dehiscence	Minor
16	74	M	26.4	—	Hindfoot	Hindfoot procedure	90	2	N	—	No complication
17	70	M	28.1	DM	Midfoot	Lisfranc ORIF	—	1	N	—	No complication
18	59	F	29.2	—	Ankle	Ankle procedure	80	2	N	—	No complication
19	67	M	31.6	Obese (BMI 31.6); CAD on dual antiplatelet	Ankle	TAR	150	2	Y	Device reaction (blistering POD 20)	Minor
20	76	F	40.0	Smoking (former); obese (BMI 40.0)	Ankle	Ankle procedure	118	2	Y	Dehiscence; Deep SSI	Major
21	21	M	20.9	—	Midfoot	Midfoot procedure	—	1	Y	Superficial SSI	Minor
22	42	M	34.5	Chlorhexidine prep allergy; obese (BMI 34.5)	Midfoot	Lisfranc ORIF	52	2	Y	Dehiscence; Deep SSI	Major
23	69	M	27.9	—	Ankle	TTC fusion	118	1	N	—	No complication
24	77	M	22.1	Smoking (former)	Midfoot	Arthrodesis	27	1	N	—	No complication
25	14	M	51.0	Obese (BMI 51.0)	Hindfoot	Revision	64	1	Y	Dehiscence; device reaction	Minor
26	53	F	48.0	DM; obese (BMI 48.0)	Hindfoot	Hindfoot procedure	0	1	Y	Dehiscence	Healing
27	23	M	48.5	Obese (BMI 48.5); contralateral AKA	Ankle	TTC fusion	108	1	N	—	No complication
28	67	M	32.0	Obese (BMI 32.0); adhesive allergy	Ankle	TAR	160	2	N	—	No complication
29	27	M	35.3	Spastic diplegic CP; obese (BMI 35.3)	Ankle	TTC fusion	134	1	N	—	No complication

Abbreviations: BMI, body mass index; CP, cerebral palsy; DM, diabetes mellitus; ED, emergency department; FMTB, force-modulating tissue bridge; ORIF, open reduction and internal fixation; POD, postoperative day; SLE, systemic lupus erythematosus; SSI, surgical site infection; TAR, total ankle replacement; TQ, tourniquet; TTC, tibiotalocalcaneal.

a Patient identifiers removed for confidentiality. Comorbidities were summarized; see Methods for complete definitions. All 29 patients have completed 90 days or more of follow-up.
